# Phosphorylation-Dependent Protein Interaction with *Trypanosoma brucei* 14-3-3 Proteins that Display Atypical Target Recognition

**DOI:** 10.1371/journal.pone.0015566

**Published:** 2010-12-21

**Authors:** Masahiro Inoue, Kouichi Yasuda, Haruki Uemura, Natsumi Yasaka, Hiroshi Inoue, Yoshitatsu Sei, Nobuo Horikoshi, Toshihide Fukuma

**Affiliations:** 1 Division of Eukaryotic Microbiology, Department of Infectious Medicine, Kurume University School of Medicine, Kurume, Japan; 2 Department of Protozoology, Institute of Tropical Medicine, Nagasaki University, Nagasaki, Japan; 3 Gastroenterology Section, Digestive Disease Branch, National Institute of Diabetes, Digestive and Kidney Diseases, Bethesda, Maryland, United States of America; 4 Division of Molecular Radiation Biology, Department of Radiation Oncology, University of Texas Southwestern Medical School, Dallas, Texas, United States of America; Louisiana State University, United States of America

## Abstract

**Background:**

The 14-3-3 proteins are structurally conserved throughout eukaryotes and participate in protein kinase signaling. All 14-3-3 proteins are known to bind to evolutionally conserved phosphoserine-containing motifs (modes 1 and/or 2) with high affinity. In *Trypanosoma brucei*, 14-3-3I and II play pivotal roles in motility, cytokinesis and the cell cycle. However, none of the *T. brucei* 14-3-3 binding proteins have previously been documented.

**Methodology/Principal Findings:**

Initially we showed that *T. brucei* 14-3-3 proteins exhibit far lower affinity to those peptides containing RSx*pS*xP (mode 1) and RxY/Fx*pS*xP (mode 2) (where x is any amino acid residue and *pS* is phosphoserine) than human 14-3-3 proteins, demonstrating the atypical target recognition by *T. brucei* 14-3-3 proteins. We found that the putative *T. brucei* protein phosphatase 2C (PP2c) binds to *T. brucei* 14-3-3 proteins utilizing its mode 3 motif (–*pS*/*pT*x_1-2_-COOH, where x is not Pro). We constructed eight chimeric PP2c proteins replacing its authentic mode 3 motif with potential mode 3 sequences found in *Trypanosoma brucei* genome database, and tested their binding. As a result, *T. brucei* 14-3-3 proteins interacted with three out of eight chimeric proteins including two with high affinity. Importantly, *T. brucei* 14-3-3 proteins co-immunoprecipitated with an uncharacterized full-length protein containing identified high-affinity mode 3 motif, suggesting that both proteins form a complex *in vivo*. In addition, a synthetic peptide derived from this mode 3 motif binds to *T. brucei* 14-3-3 proteins with high affinity.

**Conclusion/Significance:**

Because of the atypical target recognition of *T. brucei* 14-3-3 proteins, no 14-3-3-binding proteins have been successfully identified in *T. brucei* until now whereas over 200 human 14-3-3-binding proteins have been identified. This report describes the first discovery of the *T. brucei* 14-3-3-binding proteins and their binding motifs. The high-affinity phosphopeptide will be a powerful tool to identify novel *T. brucei* 14-3-3-binding proteins.

## Introduction

*Trypanosoma brucei* is the causative agent of sleeping sickness in man and nagana disease in cattle and one of the most divergent eukaryotes from mammals. The disease is spread by the tsetse fly, in which the procyclic forms (PCF) proliferate and differentiate into bloodstream forms (BSF), the life stage that then proliferates in the mammalian host. The disease is fatal if left untreated and no effective drug is currently available for treatment of the late stage of the disease (i.e., involvement of the central nervous system).

The 14-3-3 proteins are highly conserved dimeric acidic proteins acting as phosphoserine/phosphothreonine-dependent chaperones [1], [2]. Homologues of 14-3-3 proteins have been found in all eukaryotes [3], [4]. Every organism expresses at least one 14-3-3 protein that binds to phosphopeptides containing consensus motifs (mode 1 and/or mode 2) with high affinity (nanomolar levels). The motifs include both RSx*pS*xP (mode 1) and RxY/Fx*pS*xP (mode 2) where *pS* is phosphoserine [5], and the recently identified –*pS*/*pT*x_1-2_-COOH (mode 3) where x is not Pro [6]. Only limited number of proteins are known to have the mode 3 motif [7]. 14-3-3 proteins also have the ability to bind other than the modes 1-3 motifs [8], [9], [10], [11]. The latest bioinformatic and experimental survey of 14-3-3-binding sites reveal that alternative mode 1 Rxx(pS/pT)xP motifs dominate, although the last Pro occurs less than half [12]. When 14-3-3 proteins bind to their partners, the interacting partners may change their intracellular localization, preference of interacting partners, or enzymatic functions through conformational changes or masking of the functional amino acid residues [8], [9], [10], [11]. In mammalian cells, the characterization of signal transduction pathways involving kinase/phosphatase has progressed extensively through the discovery of more than 200 14-3-3-interacting proteins, mainly mediated by phosphorylated serine/threonine residue(s) of the target proteins [13].

There is still a gap in our understanding of signal transduction pathways in protozoan parasites including *T. brucei*. Although we have previously reported that both *T. brucei* 14-3-3I and II proteins play important roles in cell motility, cytokinesis and the cell cycle [14], phosphoserine-dependent *T. brucei* 14-3-3-interacting proteins have not been found until now in spite of extensive efforts. Therefore, we examined the differences between human 14-3-3 isoforms and *T. brucei* 14-3-3 isoforms with respect to affinities to various ligands. Here we provide several lines of evidence that the 14-3-3I, and especially the II, isoforms bind far less efficiently to the conventional consensus motifs (modes 1 and 2). In addition, heterodimerized 14-3-3I and II, the major existing forms in vivo ([14] and unpublished data), showed detectable affinities to the chimeric proteins containing the mode 3 motif, leading us to identify the *T. brucei* 14-3-3 binding proteins. The overall data highlight the scarcity of 14-3-3 target proteins with high affinity in the *T. brucei* cells and may indicate the divergent roles of *T. brucei* 14-3-3 proteins. The newly identified phosphopeptide that binds to *T. brucei* 14-3-3 proteins may be utilized in isolating a novel class of *T. brucei* 14-3-3 binding proteins, since over 200 human 14-3-3-binding proteins can be purified from HeLa cell extracts by a competitive elution from 14-3-3 affinity columns with alternative mode 1 phosphopeptide or high-affinity peptide antagonist of 14-3-3 proteins [13], [15].

## Results and Discussion

### *T. brucei* 14-3-3 proteins only weakly bind with c-Raf and conventional consensus phosphopeptides

**Figure 1:**
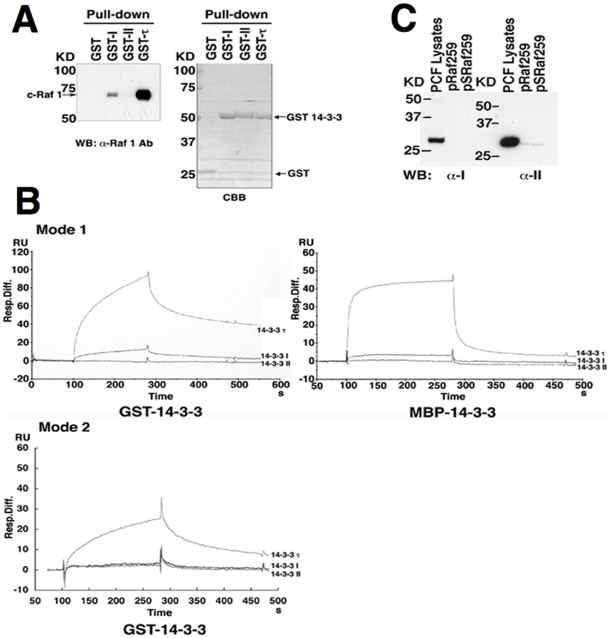
Interactions of *T. brucei* 14-3-3 proteins with c-Raf and the 14-3-3 binding phosphopeptide. (A) Interaction of GST-I, -II and τ with human c-Raf. HeLa cell lysates were subjected to GST pull-down assay using the indicated proteins bound to glutathione beads. Bound proteins were separated with SDS-PAGE, transferred to a PVDF membrane and detected with α-human c-Raf-1 antibodies (WB). The membrane was stained with coomassie brilliant blue (CBB) to visualize GST-14-3-3 proteins used in the assays. (B) Surface plasmon resonance measurements. Surface plasmon resonance analysis of interaction of c-Raf-derived phosphopeptide pSRaf259 (biotin-MAGGGRQRST*pS*TPN) (mode 1 peptide) to GST- and/or MBP-14-3-3 fusion proteins. Surface plasmon resonance analysis of a mode 2 peptide (biotin-MAGGGRLYH*pS*LP) to GST-14-3-3 fusion proteins. Sensorgram patterns are shown in each injected protein. (C) Peptide pull-down assay. *T. brucei* PCF cell lysates (5×10^8^ cells/pull-down) were subjected to peptide pull-down assay followed by Western blotting (WB) with α-I or –II antibodies. WB of total cell lysates (1×10^7^ cells) using α-I and -II detected bands of 28 kDa and 30 kDa, respectively.

Amino acid sequences of 14-3-3 proteins responsible for monomer stabilization, dimer formation and serine/threonine-phosphorylated motif binding are well conserved throughout the eukaryotes [8], [9], [10], [11]. The critical amino acid residues, with the exception of those responsible for dimer formation [9], [16], [17] are also conserved in *T. brucei* [14]. The high conservation of sequences makes yeast 14-3-3 genes to be genetically exchangeable with those of plants and mammals [18], and these 14-3-3 proteins bind to human c-Raf 1 [18]. In addition, c-Raf 1 possesses at least four 14-3-3 binding sites, namely Ser-259, Ser-621 and Ser-233, as well as a site located in the Cys-rich domain between residues 136 and 187 [10]. Therefore, glutathione S-transferase (GST) pull-down assay was carried out using HeLa cell lysates to examine whether *T. brucei* 14-3-3I and/or II may also interact with human c-Raf 1. The results showed that GST-14-3-3I bound weakly to c-Raf 1 in comparison to human GST-14-3-3τ and also that GST-14-3-3II did not bind to c-Raf 1, suggesting that *T. brucei* 14-3-3I and II do not have high affinities to human c-Raf 1 in spite of the presence of the conserved putative structure of amphipathic groove (Figure 1A and Figure S1). Thus, surface plasmon resonance analysis utilizing BIAcore was performed to determine the affinities to the conserved phosphopeptides. The mode 1 (RSx*pS*xP) and mode 2 (RxY/Fx*pS*xP) phosphopeptide motifs are present in many mammalian 14-3-3 binding proteins [5] and mode 3 (–*pS*/*pT*x_1-2_-COOH) has been identified recently [6]. All 14-3-3 proteins so far identified have high affinity to these peptide motifs [5], [6], [8], [9], [10], [11]. However, the binding patterns of the sensorgram shown in Figure 1B indicate that both maltose-binding protein (MBP)-14-3-3I and GST-14-3-3I bind only weakly (slow association and fast dissociation) to c-Raf-derived phosphopeptide pSRaf259, a representative of mode 1 phosphopeptide, compared with MBP/GST-14-3-3τ, and MBP/GST-14-3-3II did not show any interaction (Figure 1B, Mode 1, GST-14-3-3, MBP-14-3-3). We also examined the interaction of mode 2 phosphopeptide (biotin-MAGGGRLYH*pS*LP) with GST-14-3-3 proteins. The patterns of the sensorgram indicated that GST-14-3-3τ bound to the mode 2 phosphopeptide but neither GST-14-3-3I nor GST–14-3-3II showed any binding (Figure 1B, Mode 2). Comparison of mode 1 and mode 2 sensorgram data showed a slightly higher affinity of GST-14-3-3τ with mode 1 than mode 2 peptide (Figure 1B). We calculated the dissociation constant (Kd) of the mode 1 phosphopeptide by using the BIAcore program (BIAevaluation 3.0). The estimated Kd of 14-3-3I was 4.8×10^−5^ whereas that of 14-3-3τ was 2.2×10^−7^. Interaction of 14-3-3II with the mode 1 phosphopeptide was undetectable under these experimental conditions. We also investigated the interactions of endogenous 14-3-3I and II with the peptides pRaf259 and pSRaf259, in which 259Ser are unphosphorylated and phosphorylated, respectively. The peptide pull-down assay using *T. brucei* PCF lysates showed that endogenous 14-3-3I and 14-3-3II did not interact with either pRaf259 or pSRaf259 (Figure 1C). Longer exposure revealed a weak interaction for 14-3-3II with both pRaf259 and pSRaf259: the amount of captured 14-3-3II was not different between phospho-(pSRaf259) and non-phospho-(pRaf259) peptide-conjugated beads. No 14-3-3I was recovered by the peptide-conjugated beads. These results indicate that *T. brucei* 14-3-3 proteins possess far less affinity to the typical 14-3-3 binding motifs than 14-3-3 proteins from other species. Since all the essential amino acid residues and sequences of α-helices required for phosphopeptide binding in the putative amphipathic groove are well conserved in *T. brucei* 14-3-3 (Figure S1), we speculate a minor structural difference(s) or distinctive sequences of N- and/or C- termini might influence the affinity to the phosphopeptides.

### The target proteins for *T. brucei* 14-3-3 interaction

Mammalian or yeast 14-3-3 proteins have been successfully used as probes in far-Western blot (Far-WB) to identify direct interactions with numerous target partners [13], [15], [19], although the Far-WB assay has certain limitations related to the conformational state of the protein. Therefore, we used far-Western blot (Far-WB) analysis to search for binding proteins of *T. brucei* 14-3-3. Lysates were prepared from HeLa and *T. brucei* PCF cells treated with or without calyculin A (CalA), a serine/threonine phosphatase inhibitor, to increase the number and amount of phosphorylated proteins [13], [20]. None of the proteins, except 14-3-3 isoforms including putative dominant negative forms (I K77E and II K56E) that correspond to human 14-3-3ζ K49E and 14-3-3τ K49E, respectively, interacted with *T. brucei* 14-3-3I and II as shown the bands of approximately 28–30 kDa (Figure S1) [8]. The 14-3-3τ proteins interacted with various proteins including isoforms of human 14-3-3 proteins (Figure S2A). The number and amounts of the 14-3-3τ-interacting protein in both PCF and HeLa cells increased upon CalA treatment (Figure S2A, GST-τ), as reported previously [13]. However, they were much less in PCF cells than those in HeLa cells when human 14-3-3τ protein was used as a probe (Figure S2A, GST-τ), suggesting that molecular recognition of 14-3-3 proteins may co-evolve with their ligands. In Arabidopsis, some isoforms of 14-3-3 increase the phosphoserine-dependent target binding in the presence of divalent cations [21]. Therefore, we carried out Far-WB analysis in the presence of 1 mM CaCl_2_. However, no major difference in the interacting protein was observed between CalA (−) and (+) in the presence or absence of calcium (Figure S2B), indicating that the binding of *T. brucei* 14-3-3 to phosphopeptide-containing proteins is Ca^2+^-independent. The slight differences between Figure S2A (GST-II) and Figure S2B (GST-II) are due to differences in the concentration of the probes (1 µg/ml in Figure S2A, 2 µg/ml in Figure S2B). The two minor bands (∼66 and 70 KD) in the blots incubated with GST alone were the non-specific bands from anti-GST antibodies (Figure S2). Similar results were obtained when digoxigenin-labeled non-fusion recombinant 14-3-3 proteins were used as probes (data not shown), suggesting that the failure of bindings of 14-3-3I and II is not due to the steric hindrance of GST moiety. These results are consistent with the results of the GST pull-down assay and the surface plasmon resonance analysis. We have also used the heterodimerized form of GST-14-3-3II +I (described in the next section) as a probe in Far-WB analysis and obtained similar results (data not shown). Taken together, our results strongly support the notion that *T. brucei* 14-3-3 proteins do not interact with the majority of human 14-3-3-binding proteins as other 14-3-3 proteins from other eukaryotic organisms do.

### Identification of *T. brucei* 14-3-3 binding protein with high affinity

Since *T. brucei* 14-3-3 did not show high affinity to the mode 1 or 2 peptide, we searched the *Trypanosoma brucei* genome database (GeneDB: http://www.genedb.org/genedb/tryp/index.jsp) for the potential binding partners of *T. brucei* 14-3-3 proteins containing a motif of –*pS*/*pT*x_1-2_-COOH (mode 3: where x is not Pro). We first used Motif Search to extract the sequences containing the mode 3 motif and then selected for the sequences that could be phosphorylated by AGC kinase (PKA, PKG, PKC or related kinases). We selected the putative AGC substrate sequences, since AGC kinase are known to mediate diverse and important cellular functions in mammalian cells. The selected sequences were listed in Table 1.

**Table 1:** Putative mode 3 motif sequences possibly phosphorylated by AGC kinase.

Peptide sequence	Gene ID	Protein Features
-WLTRSRSLW	Tb927.7.4020	Protein phosphatase 2C, putative (PP2C)
-RRRNSV	Tb09.211.0210	Atypical dual specificity phosphatase, putative
-KSKGKSG	Tb927.4.3840	Nucleolar protein, putative
-VRVKTI	Tb927.3.1490	Leucine-rich repeat protein (LRRP), putative
-RRLRSN	Tb927.6.4390	Kinesin, putative
*-RSRSRRV	Tb09.160.5130	Hypothetical protein, conserved
-IRCRTF	Tb927.8.7020	Metallo-peptidase, Clan ME, Family M16
–EHRKSSIG	Tb11.52.0001	Acyl-CoA binding protein, putative
-KRRRSV	Tb10.70.2780	Predicted SAP domain protein (p31-SAP)
-LIEGRKQTVG	Tb927.8.2520	Acetyl-CoA synthetase, putative (ACS)

*This sequence is not –*pS*/*pT*x_1-2_-COOH, but when C-terminal V is removed in vivo, it becomes –*pS*/*pT*x_1-2_-COOH.

**Figure 2:**
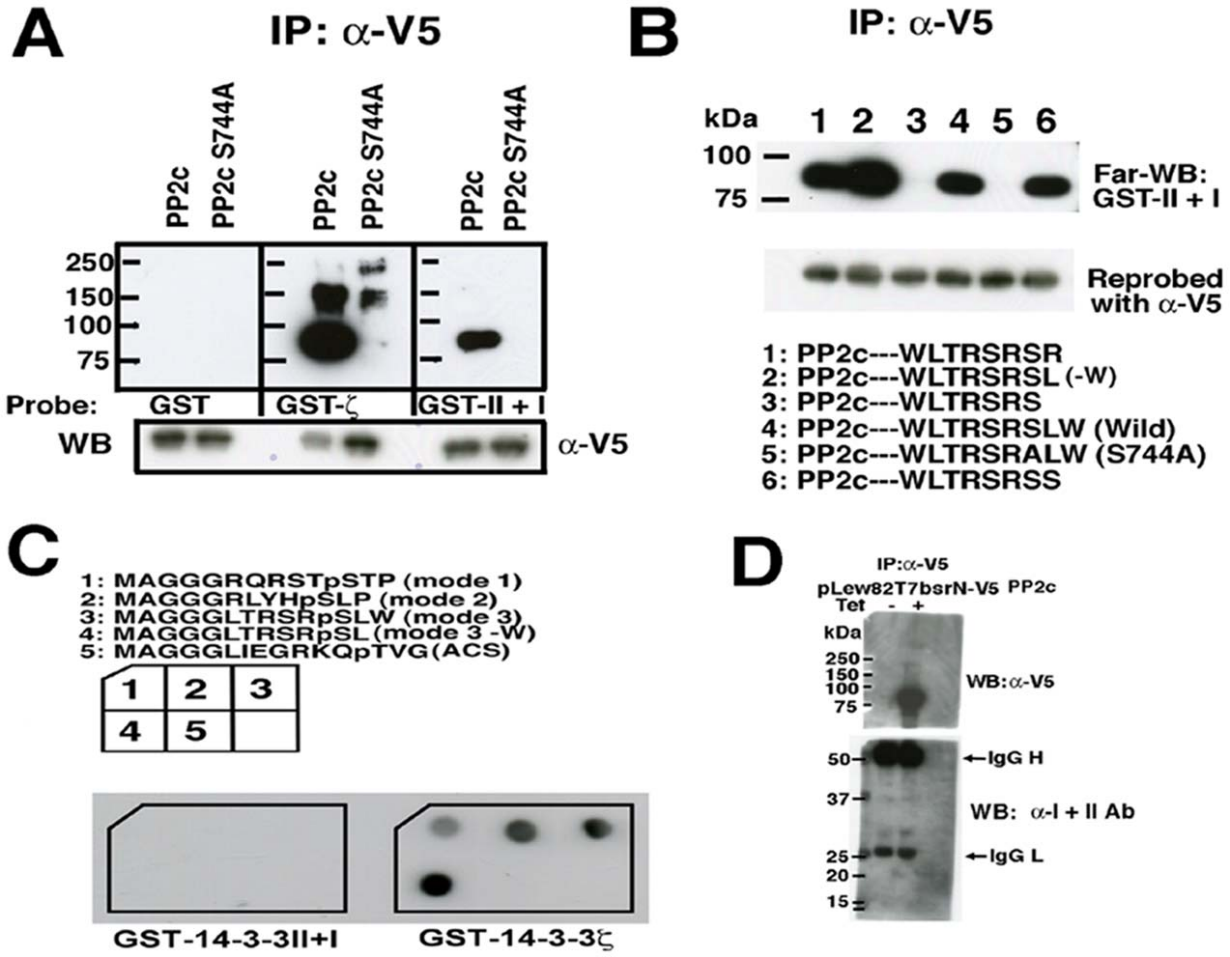
Interaction of *T. brucei* PP2c with *T. brucei* and human 14-3-3 proteins. (A) and (B) Mammalian expression vectors pCR3 N-V5-PP2c, –PP2c S744A or -C-terminal mutants of PP2c were transiently transfected to HEK293T cells. The cell lysates were subjected to immunoprecipitation with α-V5 Ab followed by far-Western blot analyses (Far-WB) using indicated probes (upper panel). Immunoprecipitated proteins were reprobed with α-V5 Ab (lower panel). (C) Modes 1-3 phosphopeptides summarized in Table 1 have far less affinity to *T. brucei* 14-3-3 than human 14-3-3ζ proteins. Indicated biotinylated phosphopeptides were mixed with streptavidine and spotted to nitrocellulose filters and dried. The filters were then incubated with indicated GST-14-3-3 proteins and detected with α-GST antibodies. (D) Tet-inducible *T. brucei* expression vector, pLew82 T7bsr-N-V5-PP2c was transfected and clones were isolated with blasticidin selection. Tet-uninduced (Tet−) or –induced (Tet +) cell lysates of the clone were subjected to immunoprecipitation followed by Western blotting using a mixture of α-I and II Ab (lower panel), and horseradish peroxidase (HRP)-labeled α-V5 Ab (upper panel).

We first examined whether a putative protein phosphatase 2c (PP2c) in the list interacts with *T. brucei* 14-3-3 proteins, since the phenotype of knockdown of 14-3-3I and/or II resembled that of okadaic acid, a potent serine/threonine phosphatase inhibitor, -treated *T. brucei* cells and PP2c has a putative mode 3 motif [22]. The N-terminally V5-tagged PP2c protein was expressed in human HEK293T cells and purified by immunoprecipitation using anti-V5 monoclonal antibody (Ab). Far-WB probing with GST-14-3-3 proteins was used to detect the direct interaction. We have recently identified the heterodimeric form as the major form of *T. brucei* 14-3-3 proteins (unpublished data) and thus, recombinant heterodimeric GST-II +I was used as a probe. The results showed that V5-tagged PP2c (but not S744A mutant protein which has Ala instead of Ser in the mode 3 motif), directly bound to GST-14-3-3ζ with high affinity and to *T. brucei* GST-II +I (heterodimerized recombinant proteins) with lower affinity (Figure 2A upper panel). We further confirmed that the binding was mediated by intact mode 3 motif (Figure 2B). Of note, PP2c mutant without W at the C-terminal end (-W) showed much higher affinity to dimeric *T. brucei* 14-3-3 proteins than wild type PP2c (Figure 2B, lane 2).

We next examined whether *T. brucei* 14-3-3 proteins recognize mode 1, 2 and 3 synthetic peptides spotted on a nitrocellulose filter. Heterodimeric *T. brucei* 14-3-3 proteins failed to show the interactions with these phosphopeptides (Table 1) including PP2c and PP2c (-W), whereas human 14-3-3ζ showed strong interactions (Figure 2C). The binding of *T. brucei* 14-3-3 proteins with PP2c might require an additional sequence(s) to establish a stable interaction. The other putative mode 3 synthetic phosphopeptides derived from ACS (Table 1) would not interact either *T. brucei* 14-3-3 or human 14-3-3ζ (Figure 2C). We next tested the association of ectopically expressed V5-tagged PP2c with endogenous 14-3-3I and II proteins in a Tet-inducible V5-tagged PP2c-expressing *T. brucei* PCF clone. Immunoprecipitation with V5 monoclonal Ab followed by Western blotting with a mixture of α-14-3-3I and II Ab failed to show the interaction of 14-3-3 with PP2c (Figure 2D lower panel), suggesting that the interaction detected by Far-WB is not stable enough to detect by immunoprecipitation. Furthermore, knockdown of PP2c gene did not affect the morphology and the growth of the *T. brucei* PCF cells (data not shown), suggesting that PP2c may not be a physiological target for *T. brucei* 14-3-3 proteins *in vivo*.

**Figure 3:**
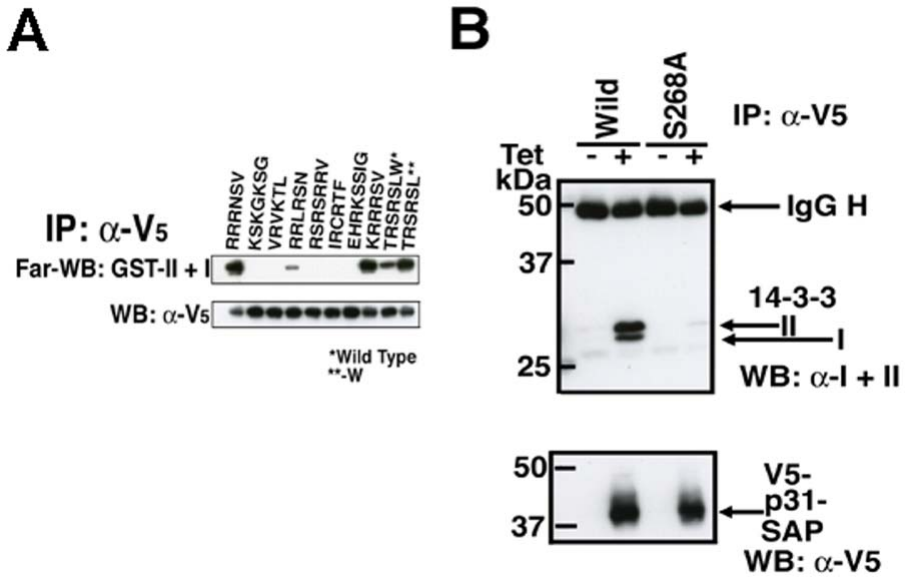
p31-SAP binds to *T. brucei* 14-3-3 proteins *in vivo* (PCF cells). (A) C-terminal chimeric PP2c proteins were produced by transfection of HEK293T cells and purified by immunoprecipitation with α-V5 Ab. Immunoprecipitated proteins were analyzed by Far-WB with GST-II + I as a probe (upper panel). Immunoprecipitated V5-tagged proteins were visualized with α-V5 Ab (lower panel). [*PP2c, **PP2c W (-)] (B) Full-length V5-tagged p31-SAP or –SAP (S286A)-expressing cell lines under the control of tetracycline (Tet) were established. Cell lysates of clones with (+) or without (-) Tet-induction were subjected to immunoprecipitation with α-V5 Ab followed by Western blotting (WB) with a mixture of α-14-3-3I and II Abs (upper panel). Western blotting with HRP-labeled α-V5 monoclonal Ab (lower panel, WB: α-V5) serves as the immunoprecipitation control. Associated 14-3-3 proteins with V5-tagged p31-SAP, and IgG heavy chain (H) are indicated with arrows (upper panel) and precipitated total V5-p31-SAP or –SAP (S286A) proteins are indicated (lower panel).

**Figure 4:**
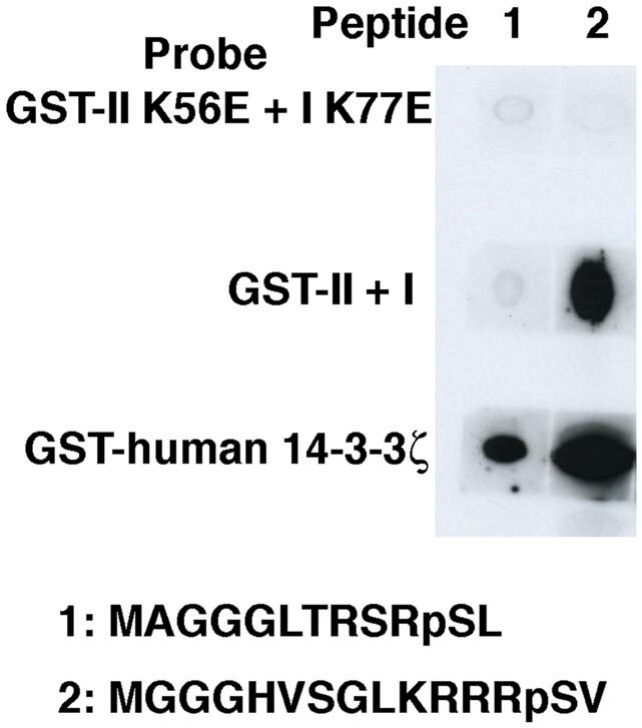
Identification of the high-affinity mode 3 phosphopeptide derived from p31-SAP. Indicated biotinylated mode 3 phosphopeptides (1 and 2) as indicated were mixed with streptavidine and spotted to nitrocellulose filters and dried. The filters were then incubated with the indicated GST-14-3-3 proteins and detected with α-GST antibodies.

To identify 14-3-3 interacting proteins containing a mode 3 motif with higher affinity than PP2c, we constructed chimeric molecules by replacing the C-terminal end of PP2c with various sequences of mode 3 motif found in the database (Table 1, except for ACS sequence). Those chimeric proteins were expressed in HEK293T cells and purified by immunoprecipitation, and the affinity to GST-II +I was compared with wild type PP2c by Far-WB analyses. Chimeras containing -RRRNSV (Tb09.211.0210) and -KRRRSV (Tb10.70.2780: predicted SAP domain protein termed p31-SAP in this manuscript) associated with GST-II +I more tightly than wild type PP2c (Figure 3A, upper panel). In order to examine whether p31-SAP binds to *T. brucei* 14-3-3 proteins through the mode 3 motif *in vivo*, Tet-inducible V5-tagged p31-SAP or p31-SAP S286A (mode 3 motif mutant)-expressing *T. brucei* PCF clones were established. Tet-induced or uninduced cell lysates were subjected to immunoprecipitation with α-V5 monoclonal Ab followed by Western blotting with a mixture of α-I and α-II-specific Ab (Figure 3B, upper panel). Western blotting with α-V5 monoclonal Ab (Figure 3B, lower panel) serves as the immunoprecipitation control. Importantly, p31-SAP but not p31-SAP S286A, a mode 3 mutant, binds to 14-3-3I and II *in vivo* (PCF cells) suggesting that the interaction is mediated by the mode 3 motif (Figure 3B). We then examined whether the p31-SAP- derived mode 3 phosphopeptide interact with *T. brucei* 14-3-3 proteins. The synthetic phosphopeptide MGGGHVSGLKRRRpSV derived from p31-SAP was clearly detected by heterodimeric *T. brucei* 14-3-3I+II proteins whereas MGGGLTRSRpSL dereived from PP2c-W was not detected as demonstrated previously (Figure 2C, Figure 4). The human 14-3-3ζ interacts with both phosphopeptides but with the preferential binding to the mode 3 peptide derived from p31-SAP (Figure 4). The human 14-3-3ζ shows slightly higher affinity to the p31-SAP derived phosphopeptide than *T. brucei* 14-3-3 (Figure 4). Overall data suggest that human 14-3-3 proteins have higher affinity to all the binding motifs used in our experiments than *T. brucei* 14-3-3 proteins. In addition, mutations in the putative critical amino acid residues in both 14-3-3I (K77E) and II (K56E) (Figure S1) prevent the binding to the p31-SAP derived peptide (Figure 4, GST-I K77E +II K56E), suggesting that the structure of an amphipathic groove that mediate the association of 14-3-3 proteins with phosphopeptides are evolutionally conserved between far distant organisms. Subtle difference(s) in the structure of the amphipathic groove or the distinctive difference(s) in the structure of the N- and/or C-termini, may affect the binding to the motifs. Since no phosphopeptide was known to interact with *T. brucei* 14-3-3 proteins until now, the affinity purification method eluting with a specific phosphopeptide or a high-affinity peptide antagonist of 14-3-3 proteins, which is successfully employed to isolate a great number of 14-3-3-interacting proteins in other organisms [13], [15], has not been possible. The newly identified high-affinity phosphopeptide (HVSGLKRRRpSV) is the first available phosphopeptide that can be utilized for the affinity purification of 14-3-3-binding proteins in *T. brucei* and for the subsequent identification of novel binding motifs (unpublished data).

*T. brucei* 14-3-3 proteins exhibit far lower affinity to the evolutionally conserved consensus binding motifs (modes1 and 2) and slightly lower affinity to the newly identified mode 3 sequence when compared to those in human 14-3-3 proteins, representing the atypical nature of *T. brucei* 14-3-3 proteins. Considering the affinity to the phosphopeptides, *T. brucei* 14-3-3 proteins might not only act as phosphoserine-dependent binding proteins but also act as binding proteins utilizing hitherto unknown consensus motifs. Thus, the functions of 14-3-3 proteins in protozoan organisms such as *trypanosomatids*, the most divergent eukaryote from mammals, may need to be reconsidered. Further investigation of the atypical nature of *T. brucei* 14-3-3 proteins and the unidentified binding proteins may help identify novel drug targets since 14-3-3 proteins are essential to the survival of the parasite [14].

## Materials and Methods

### Construction of GST-I and II mutants

Mutations were made using the following primers:

I K77E: 5′-gagaacgttatcggttcccgtcgcaa-3′, 5′-gtacgccatcgagagcaggttacg-3′; and II K56E: 5′-gaaaacgtcattggagcccgtcgt-3′, 5′-atacgcgacggagagaagattgc-3′. The sequences were confirmed.

### Construction of pLew82T7bsr N-V5 PP2c, pCR3 N-V5 PP2c

The PP2c-coding sequence (Tb927.7.4020: protein phosphatase 2C, putative) was amplified by PCR using primers 5′-atgtataccagtgttagaaagcct-3′ and 5′-gggccgcaacctgtctcctcataacat-3′. The amplified PP2c sequence was inserted into pLew82T7bsr N-V5 (pLew82 is a kind gift from Dr. George Cross) and PCR3 N-V5 (PCR3 is purchased from Invitrogen) at the HpaI site. (The pLew82 vector was modified by the insertion of annealed oligonucleotides of weak T7 promoter at the BamHI site. The sequences of oligonucleotides are as follows: 5′-GATCCTTAATACGTCTCACTATAGGGA-3′, and 5′-GATCTCCCTATAGTGAGACGTATTAAG-3′. Permanent transfectants with pLew82 T7 vector do not require 1 ng/ml Tet for maintenance of the clones, while pLew82 transfectants do. The N-terminal V-5 tag sequence and HpaI site were introduced in the pLew82T7 vector to make pLew82T7 N-V5. Drug selection maker was replaced with a blasticidin-resistance gene, which was designated as pLew82T7bsrN-V5.)

### Construction of pLew82T7bsrN-V5 p31-SAP

The P31*-*SAP-coding (Tb10.70.2780: predicted SAP domain protein) sequence was amplified by PCR using primers 5′-atgaggaaacccgggcggaaaatt-3′ and 5′-acgggtgctgataatgtaaccaa-3′. The amplified sequence was inserted in the vector. The mutants were created using PCR and the sequences were confirmed.

### Construction of C-terminal PP2C mutants

The C-terminal HpaI site in the PP2c gene and the XbaI site in the vector were used to insert the annealed oligonucleotides encoding the indicated amino acid sequences.

### Raf259 peptide pull-down assay

Ten µl of streptavidin agarose beads (Sigma Chemical Co., St. Louis, MO) were incubated with 10 µl of 100 µM of pRaf 259 (biotin- MAGGGRQRST*S*TPN) and/or pSRaf 259 at room temperature for 30 min and the beads were washed three times with lysis buffer (150 mM NaCl, 10 mM HEPES, pH 7.5, 0.2% NP-40, 50 mM NaF, 1 mM Na_3_VO_4_ and Roche protease inhibitor tablets). Next, 1×10^9^ cells of *T. brucei* PCF were lysed with 2 ml of the lysis buffer on ice. Insoluble proteins were pelleted and 1 ml of the supernatant was used for each peptide pull-down assay. The lysates were incubated with each bead, rotated at 4°C for 2 hrs and washed with the lysis buffer three times. Twenty µl of 2xSDS Gel loading buffer with 2-mercaptoethanol were added to the washed beads, and 5 µl of eluates were run on 10–20% SDS-PAGE. The blots were detected with α-I and/or α–II Abs as described previously [14].

### Production and purification of heterodimeric 14-3-3 proteins with glutathione S-transferease (GST), *GST-II +I*

The coding sequences of *T. brucei* 14-3-3I and II were subcloned into pRSFDuet-1 (Novagen) and pGEX6P1 (GE-Healthcare. Both constructs were simultaneously used to transform BL21 (T7 Express lysY/Iq competent *E. coli*, NEB). After IPTG-induction, purification was carried out using glutathione beads (GE-Healthcare). SDS-PAGE analysis of the purified proteins showed two distinctive bands, GST-14-3-3II and 14-3-3I without a peptide-tag, with equal molar ratio (data not shown).

### Surface plasmon resonance measurements

The surface plasmon resonance measurement was carried out using BIAcore 2000. A resonance unit value of 220 of biotinylated phosphopeptide pSRaf 259 (biotin-MAGGGRQRST*pS*TPN) (mode 1 peptide) was coupled to a streptavidin-coated sensor chip (SA5, BIAcore). In the next step, 200 nM each of purified GST-14-3-3I, GST-14-3-3II, and GST–14-3-3τ, or 500 nM each of MBP-14-3-3I, MBP-14-3-3II, and MBP-14-3-3τ in HBS-EP buffer (BIAcore) was injected at a flow rate of 20 µl/min. A resonance unit value of 296 of biotinylated mode 2 peptide (biotin-MAGGGRLYH*pS*LP) was also coupled to SA5 and 200 nM each of GST-14-3-3I, GST-14-3-3II and GST-14-3-3τ in HBS-EP buffer, and was injected in the same manner.

### Transfection and establishment of clones

Transfection and cloning of *T. brucei* 29-13 procyclic cells (a kind gift from George Cross) were performed as previously described [23]. Finally, 1 µg/ml of Tet was added upon induction of the genes. Transfection of SV40 large T-antigen-transformed human embryonic kidney (HEK 293T) cells (Gene Hunter Corporation) was performed using Fugene 6 transfection reagent (Roche).

### Interaction of GST-14-3-3I, -II and τ with human c-Raf

HeLa cell lysates were prepared using NP-40 lysis buffer (150 mM NaCl, 10 mM HEPES, pH 7.5, 0.2% NP-40, 50 mM NaF, 1 mM Na_3_VO_4_ and Roche protease inhibitor tablets) and subjected to GST pull-down assay. Bound proteins were separated with SDS-PAGE, transferred to a PVDF membrane and detected with anti-human c-Raf-1 antibodies (Santa Cruz Biotechnology, Santa Cruz, CA).

### Far-Western blot analysis

Far-Western blot analysis of Ser/Thr phosphatase inhibitor Calyculin A (CalA) treated + and/or untreated – of HeLa and/or 29-13 PCF cell lysates was performed. HeLa cells and/or 29-13 PCF cells were lysed with SDS-gel loading buffer supplemented with 2-mercaptoethanol (Bio-Rad, Hercules, CA) and sonicated. The cell lysates equivalent to 5×10^5^ HeLa cells and 1×10^7^ 29-13 PCF cells were applied on 4–20% SDS-PAGE (Daiichi, Japan) and transferred to nitrocellulose filters (Millipore, Bedford, MA). The filters were denatured with denaturation buffer (6M guanidine-HCl, 50 mM Tris-HCl, pH 8.0, and 1 mM dithiothreitol [DTT]). The denaturation buffer was then diluted with an equal volume of 50 mM Tris-HCl, pH 8.0 with 1 mM DTT. After 15 min of denaturation, the filters were then treated with 2x diluted denaturation buffer. The same step was repeatedly carried out eight times and then renatured filters were washed twice with TTBS (50 mM Tris-HCl, pH 7.4, 0.1% Tween 20, and 150 mM NaCl). The resultant filters were blocked with TTBS containing 4% skim milk plus 1 mM DTT. Then the filters were incubated overnight with 1 or 2 µg/ml of GST-14-3-3 probes in 4% skim milk containing the blocking solution at 4°C. The filters were washed three times with TTBS and incubated with anti-GST polyclonal antibodies (Sigma) followed by horseradish peroxidase (HRP)-labeled anti-rabbit goat IgG (Jackson Immunoresearch Laboratories, West Grove, PA).

### Immunoprecipitation

Immunoprecipitation was carried out using NP-40 lysis buffer. Anti-V5 monoclonal Ab and sepharose suspension protein G (protein G beads) were purchased from Nacalai and Invitrogen, respectively. In brief, cells were lysed on ice for 30 min and spun at 15,000 rpm for 10 min. The resultant supernatants were used for immunoprecipitation using 5 µl of protein G beads and 1 µg of α-V5 monoclonal Ab.

### NC-filter binding assay

Five µl of 1 mM biotinylated peptides, purchased from Scrum (Japan) were mixed with 15 µl of streptavidine (1 mg/ml) in the presence of 0.1% Tween20, spotted on NC-filters and dried. Filters were washed with TTBS, blocked with Protein-Free Blocking Buffer (Pierce), and then incubated with GST-14-3-3 proteins in TTBS containing 20% Protein-Free Blocking Buffer and 1 mM DTT. The filters were extensively washed four times with TTBS, and detected with anti-GST antibodies.

## Supporting Information

Figure S1 Amino acid sequence alignment of *T. brucei* 14‐3‐3I, II, human 14‐3‐3τ, and ζ. The amphipathic groove structures are comprised of α‐helices 3, 5, 7 and 9 as shown in green lines. Amino acid residues directly engaged in the conserved phospho‐peptide bindings are boxed in red. Identical amino acid residues are colored in magenta.
(TIF)

Figure S2 Far‐western blot (Far‐WB) analyses of the binding proteins for human and *T. brucei* 14‐3‐3 proteins. (A) The nature of GST‐14‐3‐3 probes, samples, and the calyculin A (CalA) treatment are indicated. (B) The effect of 1 mM CaCl_2_ on *T. brucei* 14‐3‐3 binding was determined by Far‐WB analysis. Data are representatives of three independent experiments.
(TIF)
