# Supplementary figures and images for: Phosphorylation-Dependent Protein Interaction with Trypanosoma brucei 14-3-3 Proteins that Display Atypical Target Recognition
Source: PLoS One. 2010 Dec 21;5(12):e15566. doi: 10.1371/journal.pone.0015566 (PMC3006207; doi:10.1371/journal.pone.0015566)

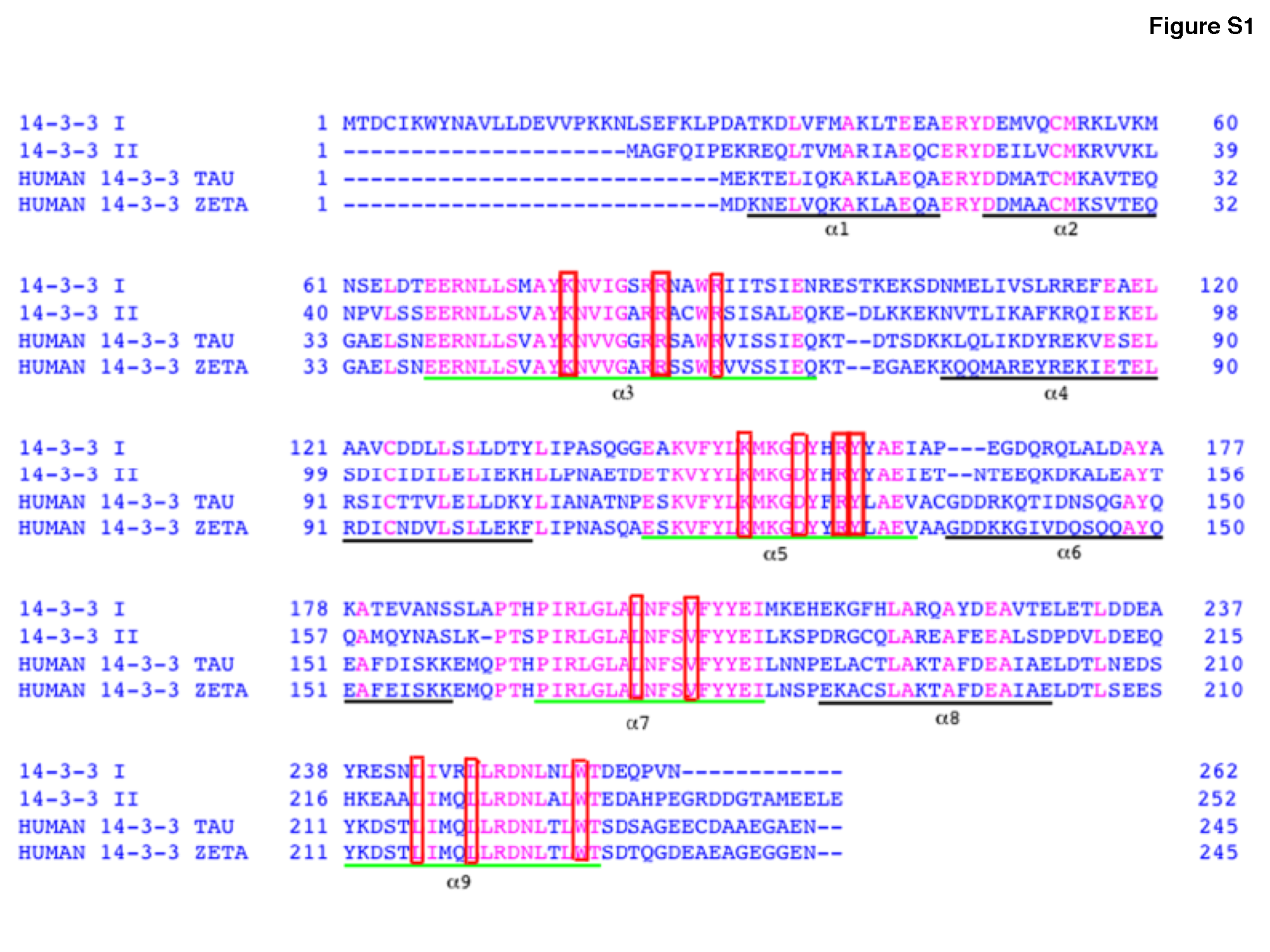

Supplement: Figure S1 — Amino acid sequence alignment of T. brucei 14‐3‐3I, II, human 14‐3‐3τ, and ζ. The amphipathic groove structures are comprised of α‐helices 3, 5, 7 and 9 as shown in green lines. Amino acid residues directly engaged in the conserved phospho‐peptide bindings are boxed in red. Identical amino acid residues are colored in magenta. (TIF) [file pone.0015566.s001.tif]

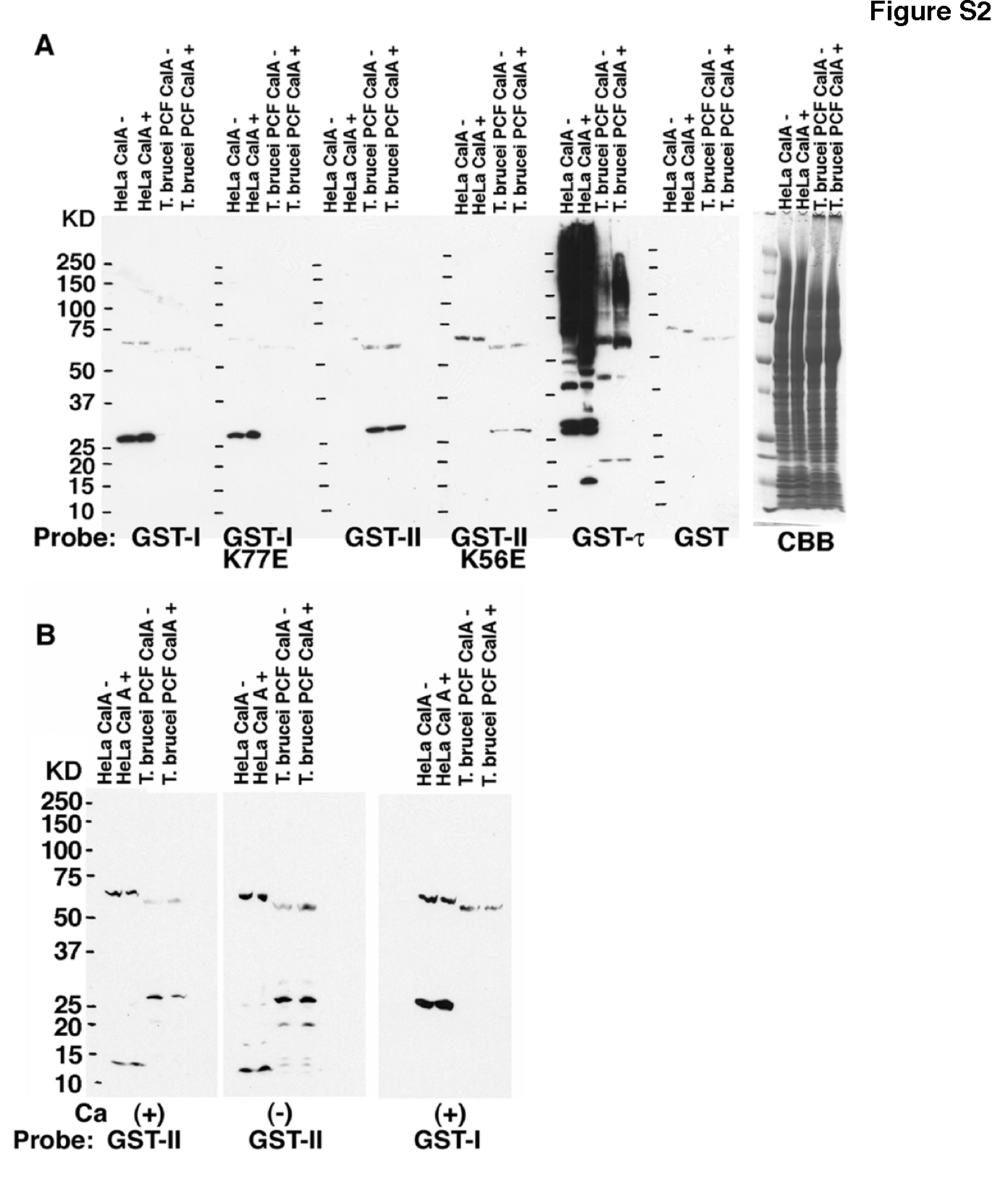

Supplement: Figure S2 — Far‐western blot (Far‐WB) analyses of the binding proteins for human and T. brucei 14‐3‐3 proteins. (A) The nature of GST‐14‐3‐3 probes, samples, and the calyculin A (CalA) treatment are indicated. (B) The effect of 1 mM CaCl2 on T. brucei 14‐3‐3 binding was determined by Far‐WB analysis. Data are representatives of three independent experiments. (TIF) [file pone.0015566.s002.tif]
